# Comparison of the Image Quality of Turbo Spin Echo and Echo-Planar Diffusion-Weighted Imaging of the Head and Neck Region

**DOI:** 10.7759/cureus.67157

**Published:** 2024-08-18

**Authors:** Ajina Sam, Iffath Misbah, Jasvant Ram, Sam Raja

**Affiliations:** 1 Department of Radiology, Saveetha Medical College and Hospital, Saveetha Institute of Medical and Technical Sciences, Saveetha University, Chennai, IND

**Keywords:** echo planar imaging diffusion-weighted imaging, distortion ratio, contrast-to-noise ratio, signal-to-noise ratio, magnetic resonance imaging of the head and neck area, turbo spin echo mri

## Abstract

Background: Magnetic resonance imaging (MRI) of the head and neck region is notably challenging due to the complex anatomy and the critical need for high-resolution imaging to accurately diagnose various pathologies. The two prominent MRI techniques used in this context are turbo spin echo (TSE) and echo-planar diffusion-weighted imaging (EP-DWI). TSE is recognized for providing high-resolution anatomical images, whereas EP-DWI offers functional imaging that highlights the diffusion of water molecules, essential for detecting early pathological changes. This study aims to compare the image quality of TSE and EP-DWI in the head and neck region to assess their diagnostic efficacy and clinical utility.

Methods: This retrospective study was conducted at Saveetha Medical College and Hospital over six months. A total of 100 patients (50 males and 50 females, aged 18-65 years) with various head and neck pathologies were included. Patients underwent both TSE and EP-DWI sequences using a Philips MULTIVA 1.5 T scanner. Image quality was assessed based on signal-to-noise ratio (SNR), contrast-to-noise ratio (CNR), artifact presence, and lesion detection. Two experienced radiologists independently reviewed the images, with inter-observer agreement calculated using Cohen's kappa coefficient.

Results: The mean SNR for TSE was significantly higher than EP-DWI (45.2 vs. 28.7, p<0.01), indicating superior image clarity and detail in TSE images. TSE demonstrated a higher mean CNR compared to EP-DWI (25.4 vs. 15.8, p<0.01), suggesting better differentiation between different tissue types and pathologies. Artifacts were more frequent in EP-DWI images (45% vs. 15%), with motion artifacts being the most common. TSE detected more lesions (120 vs. 95), with more precise delineation of lesions. The inter-observer agreement was excellent for both TSE and EP-DWI, with kappa values of 0.85 and 0.80, respectively.

Conclusion: TSE MRI provides superior image quality compared to EP-DWI for evaluating the head and neck region. The enhanced SNR and CNR in TSE images result in clearer and more detailed visualizations of anatomical structures and pathological changes, with fewer artifacts. While EP-DWI is valuable for functional imaging, its role should be complementary to TSE. The study suggests that TSE should be the preferred modality for detailed anatomical assessment in the head and neck region. Further studies with larger sample sizes and advanced imaging techniques may provide additional insights into optimizing MRI protocols for head and neck imaging.

## Introduction

Magnetic resonance imaging (MRI) of the head and neck region is notably challenging due to the complex anatomy and the critical need for high-resolution imaging to accurately diagnose various pathologies. The head and neck region is anatomically intricate, encompassing a variety of structures such as bones, muscles, glands, lymph nodes, blood vessels, and nerves. These diverse tissues require precise imaging techniques to distinguish between normal and pathological conditions effectively. The two prominent MRI techniques used in this context are turbo spin echo (TSE) and echo-planar diffusion-weighted imaging (EP-DWI). TSE is renowned for providing high-resolution anatomical images. It excels in delineating fine structural details, making it particularly useful for identifying and characterizing lesions, tumors, and other abnormalities. TSE sequences are less sensitive to magnetic field inhomogeneities, resulting in fewer artifacts and clearer images. On the other hand, EP-DWI offers functional imaging that highlights the diffusion of water molecules within tissues. This capability is essential for detecting early pathological changes, such as those seen in stroke, inflammation, and certain types of tumors, where the diffusion characteristics of water molecules can provide critical diagnostic information [1].

This study aims to compare the image quality of TSE and EP-DWI in the head and neck region to assess their diagnostic efficacy and clinical utility, thereby filling the knowledge gap regarding their comparative effectiveness in head and neck imaging. The evaluation focuses on several image quality parameters, including signal-to-noise ratio (SNR), contrast-to-noise ratio (CNR), artifact presence, and lesion detection. By understanding these factors, the study seeks to determine which technique offers better diagnostic performance and reliability, thereby aiding in the selection of the appropriate imaging modality for specific clinical scenarios. The results will have significant implications for clinical practice, guiding the selection of imaging techniques that provide the highest diagnostic yield and reliability.

The necessity for this study arises from the inherent challenges in imaging the head and neck region, compounded by the critical need for high-resolution and high-quality images to make accurate diagnoses. The head and neck region comprises a diverse array of tissues, requiring imaging techniques that can provide detailed and distinct images of these structures [2]. Accurate imaging is essential for diagnosing pathologies such as tumors, vascular anomalies, and inflammatory conditions. Artifacts, particularly motion and susceptibility artifacts, are a significant concern in MRI. Comparing TSE and EP-DWI can help identify the technique that minimizes these artifacts, thereby enhancing image clarity and diagnostic accuracy. Early detection of pathologies, especially malignant tumors and stroke, is crucial for effective treatment and better patient outcomes [3,4]. EP-DWI's ability to highlight water diffusion changes needs to be evaluated against TSE’s anatomical detail to determine which is more effective for early diagnosis. By understanding the strengths and weaknesses of TSE and EP-DWI, radiologists can optimize MRI protocols for head and neck imaging. This optimization can lead to more accurate diagnosis, better patient management, and improved outcomes.

## Materials and methods

The study was designed as a retrospective comparative analysis conducted over six months at Saveetha Medical College and Hospital. The inclusion criteria involved patients presenting with various head and neck pathologies, aged between 18 and 65 years. A total of 100 patients (50 males and 50 females) were included. Patients with contraindications to MRI, such as metallic implants or claustrophobia, were excluded from the study.

All MRI scans were performed using a Philips MULTIVA 1.5 T scanner. Each patient underwent both TSE and EP-DWI sequences as part of their imaging protocol. The TSE sequences were acquired with the following parameters: TR/TE = 4,000/100 ms, slice thickness = 4 mm, FOV = 240 mm, and matrix size = 256 x 256. For the EP-DWI sequences, the parameters were TR/TE = 4,000/80 ms, slice thickness = 4 mm, FOV = 240 mm, matrix size = 128 x 128, and b-values of 0 and 1,000 s/mm². Using a 256 x 256 matrix size in EP-DWI imaging compared to a 128 x 128 matrix size generally results in higher perceived resolution, making it more comparable to TSE images. However, the 256 x 256 EP-DWI images would likely exhibit more noise due to the smaller voxel size. Additionally, 256 x 256 matrix-size images would require longer acquisition time, increasing the likelihood of motion artifacts compared to 128 x 128 matrix-size images. These parameters were selected to optimize image quality while maintaining consistent conditions across the two imaging techniques.

The images obtained from both TSE and EP-DWI sequences were independently reviewed by two experienced radiologists who were blinded to the clinical details of the patients. Image quality assessment was based on several criteria: SNR, CNR, artifact presence, and lesion detection. These criteria were chosen to comprehensively evaluate the diagnostic efficacy and clinical utility of the two MRI techniques. SNR measures the relative signal strength of the image compared to the background noise, indicating overall image clarity. CNR measures the ability of the imaging technique to distinguish between different tissue types and pathologies, reflecting the quality of contrast in the images. The frequency and type of artifacts, particularly motion artifacts, were recorded to assess the impact on image quality. The ability to identify and characterize lesions was evaluated to determine the clinical relevance of the imaging techniques.

Inter-observer agreement was calculated using Cohen's kappa coefficient to ensure consistency and reliability of the image quality assessments. Statistical comparisons between TSE and EP-DWI were performed using paired t-tests for continuous variables, such as SNR, CNR, and average lesion size. The chi-square test was used to compare proportions, such as the presence of artifacts and the number of lesions detected. The results were summarized in tables and figures, providing a clear comparison of the key parameters between TSE and EP-DWI. The mean SNR and CNR values, the frequency of artifacts, and the success rate in lesion detection were compared, highlighting the strengths and weaknesses of each imaging technique. The statistical significance of these comparisons was reported, providing a credible basis for interpreting the findings.

The study's findings were discussed in the context of their implications for clinical practice. The superior image quality of TSE in terms of SNR and CNR, coupled with fewer artifacts, suggested its preference for detailed anatomical assessment in the head and neck region. However, the functional imaging capabilities of EP-DWI, particularly in detecting diffusion characteristics critical for diagnosing certain pathologies, were acknowledged. The study concluded with recommendations for optimizing MRI protocols for head and neck imaging, balancing the benefits of both TSE and EP-DWI to enhance diagnostic accuracy and patient outcomes.

## Results

To summarize the data collected, Table 1 provides an overview of the demographic details of the patients in the study, including total number of patients, gender distribution, age range, mean age, age distribution, common medical diagnoses, and the criteria for inclusion and exclusion. This cohort consisted of an equal distribution of male and female patients, predominantly in the middle-aged group. The range of medical conditions primarily included benign and malignant tumors, inflammatory lesions, and vascular anomalies.

**Table 1:** Demographic details of the patients in the study

Demographic Parameter	Details
Total Number of Patients	100
Gender Distribution	Males: 50
	Females: 50
Age Range	18-65 years
Mean Age	40.5 years
Age Distribution	18-30 years: 20 patients
	31-45 years: 40 patients
	46-60 years: 30 patients
	61-65 years: 10 patients
Common Medical Diagnoses	Benign Tumors: 30 patients
	Malignant Tumors: 45 patients
	Inflammatory Lesions: 15 patients (total of 25 lesions)
	Vascular Anomalies: 10 patients (total of 20 lesions)
Inclusion Criteria	Patients with various head and neck pathologies, aged between 18 and 65 years
Exclusion Criteria	Patients with contraindications to MRI, such as metallic implants or claustrophobia

Table 2 provides an overview of the key parameters and their statistical significance between TSE and EP-DWI. This reveals that TSE sequences had a significantly higher mean SNR and CNR compared to EP-DWI, indicating superior image clarity and tissue differentiation. The presence of artifacts was notably lower in TSE images, with only 15% of scans exhibiting artifacts compared to 45% in EP-DWI. The number of lesions detected was greater in TSE, and the lesions were more accurately delineated with a larger average size observed. The inter-observer agreement was excellent for both TSE and EP-DWI, with kappa values of 0.85 and 0.80, respectively.

**Table 2:** Comparison of key parameters between turbo spin echo (TSE) and echo-planar diffusion-weighted imaging (EP-DWI) The data presented in the table have been analyzed using the paired t-test for comparing the means of continuous variables (e.g., SNR, CNR, average lesion size) and the chi-square test for comparing the proportions (e.g., artifact presence, number of lesions detected). Inter-observer agreement was measured using Cohen's kappa coefficient.

Parameter	Turbo Spin Echo (TSE)	Echo Planar Diffusion-Weighted Imaging (EP-DWI)	Statistical Significance
Number of Patients	100	100	-
Mean Signal-to-Noise Ratio (SNR)	45.2 ± 5.8	28.7 ± 4.2	p < 0.01
Mean Contrast-to-Noise Ratio (CNR)	25.4 ± 4.1	15.8 ± 3.5	p < 0.01
Artifact Presence (%)	15%	45%	p < 0.01
Number of Lesions Detected	120	95	p < 0.05
Average Lesion Size (mm)	12.5 ± 3.1	10.2 ± 2.8	p < 0.05
Inter-observer Agreement (Kappa)	0.85	0.80	-

Artifacts were more frequently observed in EP-DWI images, with motion artifacts being the most common [3]. Motion artifacts were significantly more common in EP-DWI (30%) compared to TSE (10%), likely due to the longer acquisition times and greater sensitivity to patient movement in EP-DWI. Susceptibility artifacts were also more prevalent in EP-DWI (10%) compared to TSE (5%). This comparison of artifact presence between the two modalities is detailed in Table 3.

**Table 3:** Comparison of artifact presence between turbo spin echo (TSE) and echo-planar diffusion-weighted imaging (EP-DWI)

Type of Artifact	TSE (%) n=100	EP-DWI (%) n=100
Motion Artifacts	10% (n=10)	30% (n=30)
Susceptibility Artifacts	5% (n=5)	10% (n=10)
Total Artifacts	15% (n=15)	45% (n=45)

TSE detected a greater number of lesions (n=120) compared to EP-DWI (n=95). Additionally, the lesions identified by TSE were more precisely delineated, facilitating better characterization and localization [5,6]. Table 4 provides a detailed breakdown of lesion detection and average lesion sizes by pathology type, highlighting the differences between TSE and EP-DWI.

**Table 4:** Detailed breakdown of lesion detection and size by pathology The number of lesions detected in Table 4 exceeds the number of patients reported in Table 1 for some categories (e.g., inflammatory lesions, vascular anomalies). This is because some patients presented with multiple lesions of the same type.

Pathology	TSE - Lesions Detected	EP-DWI - Lesions Detected	TSE - Average Lesion Size (mm)	EP-DWI - Average Lesion Size (mm)
Benign Tumors	30	25	15.2 ± 2.8	13.1 ± 3.0
Malignant Tumors	45	40	20.3 ± 4.5	17.8 ± 4.2
Inflammatory Lesions	25	20	10.4 ± 2.1	8.9 ± 2.0
Vascular Anomalies	20	10	9.8 ± 1.5	8.1 ± 1.7

**Figure 1:**
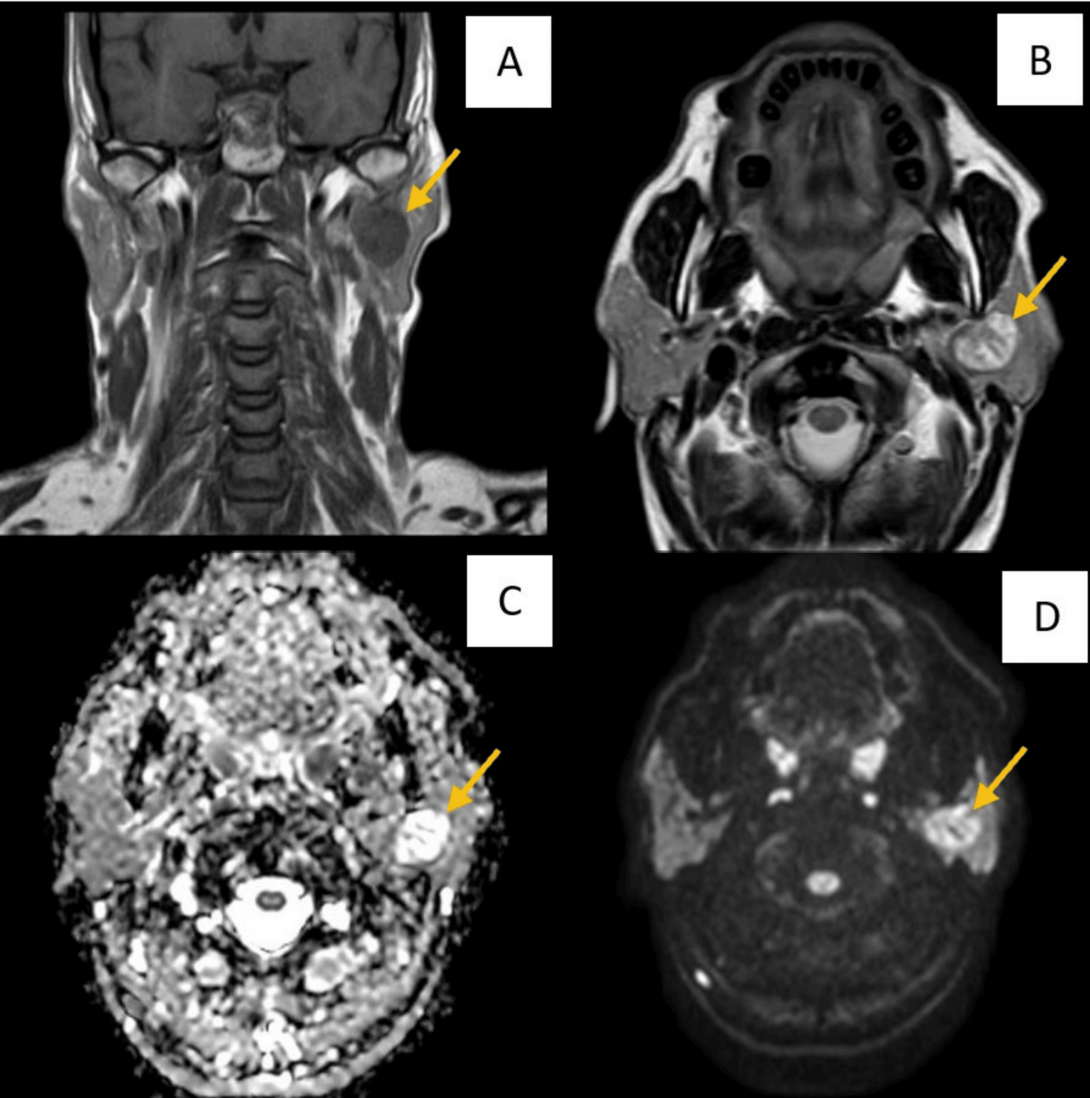
MRI images of a 55-year-old male showing primary benign neoplastic etiology involving the left parotid gland - likely Warthin's tumor A: T1-weighted imaging MRI sequence showing a well-defined hypo-intense lesion, with a lobulated margin seen in the superficial and deep lobes of the left parotid gland; B: T2-weighted imaging MRI sequence showing a well-defined hyper-intense lesion, with a lobulated margin seen in the superficial and deep lobes of the left parotid gland; C and D: ADC and DWI images showing subtle peripheral diffusion restriction

**Figure 2:**
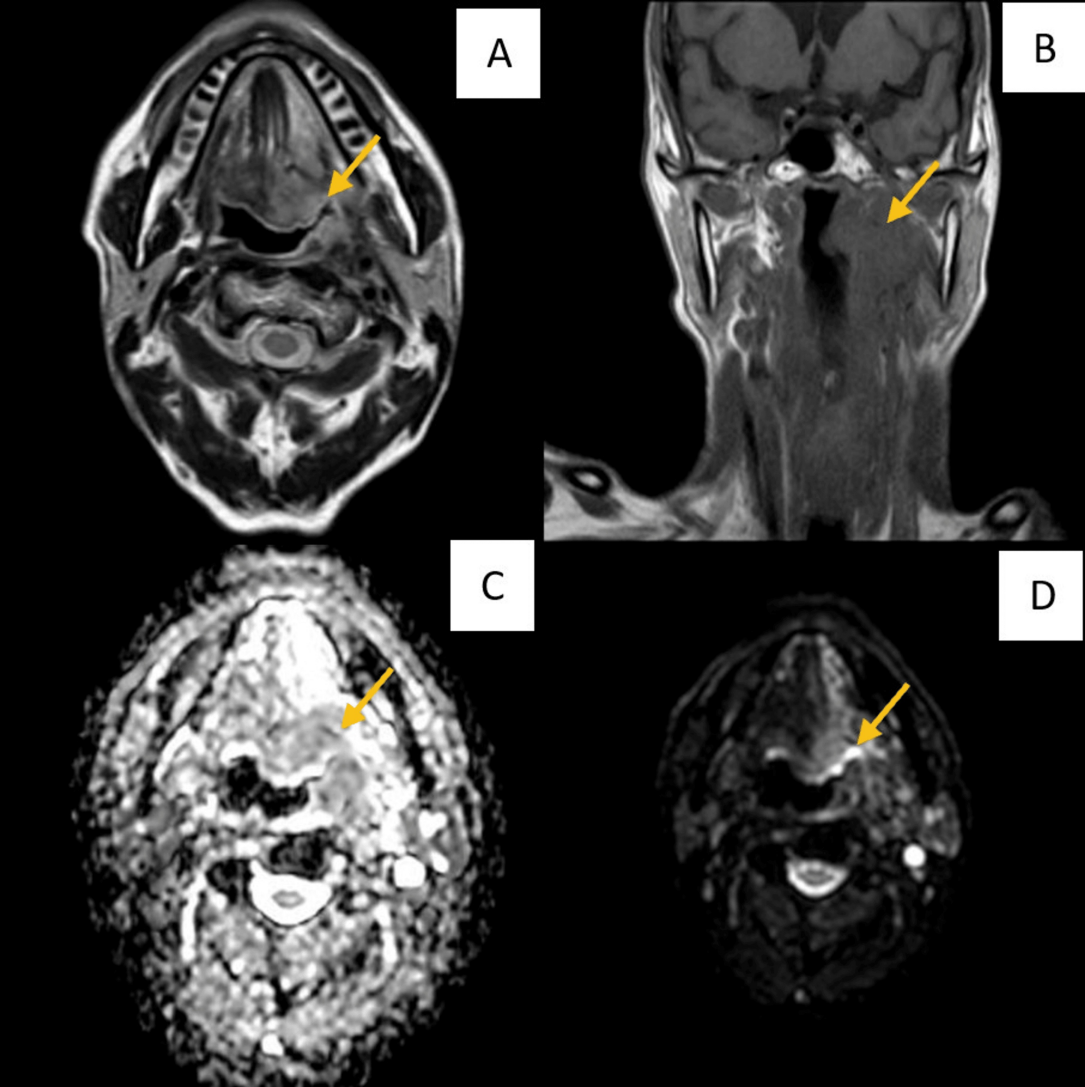
MRI images of a 57-year-old male, showing an ill-defined, diffusion restricting enhanced lesion noted in the left posterior two-third of the tongue and base of the tongue - likely malignant neoplastic etiology A: T2-weighted imaging MRI sequence showing an ill-defined T2 hyper-intense lesion seen in the left lateral aspect of the posterior two-third of the tongue and base of the tongue; B: T1-weighted imaging MRI sequence showing an ill-defined T1 iso-intense lesion seen in the left lateral aspect of the posterior two-third of the tongue and base of the tongue; C and D: Apparent diffusion coefficient (ADC) and DWI images showing diffusion restriction

**Figure 3:**
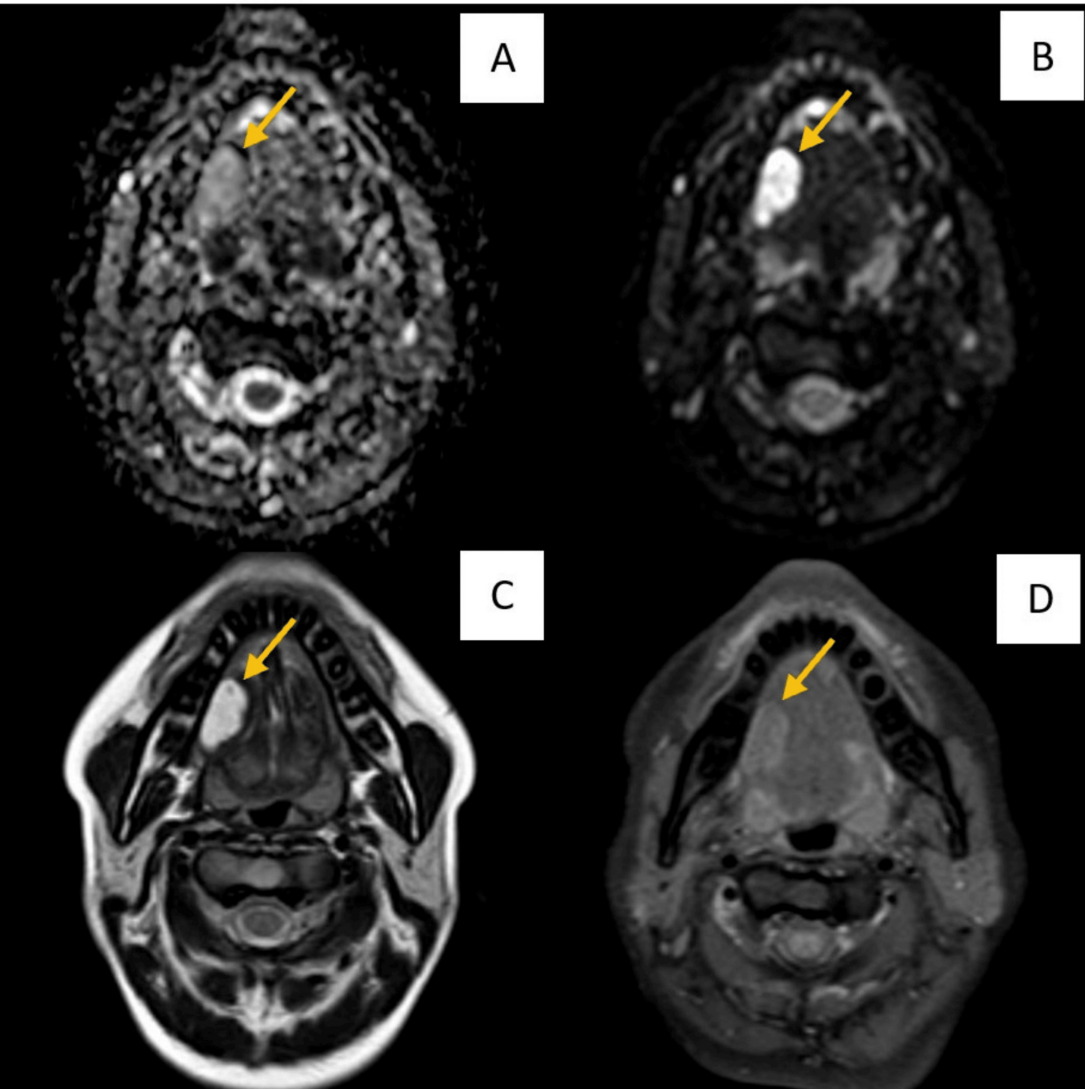
MRI images of an 18-year-old female showing mildly enhancing, fairly defined, diffusion restricting, T2 hyper-intense lesion with internal hypointensity (calcification) in the right anterior lateral aspect of the tongue involving the intrinsic and extrinsic muscles. The view of the central calcific component lesion favors hemangioma A and B: ADC and DWI showing diffusion restriction; C: T2-weighted imaging MRI sequence showing a fairly defined, T2 hyper-intense lesion with an internal hypo-intense focus is seen in the right lateral aspect of anterior 2/3rd of the tongue; D: T1-weighted imaging MRI sequence showing a fairly defined, T2 iso-intense lesion seen in the right lateral aspect of anterior 2/3rd of the tongue

The following images (Figures 1-3) show a benign, malignant pathology, and a vascular anomaly, respectively, as seen in MRI (T1, T2, DWI, and ADC) sequences.

These results highlight the advantages of TSE over EP-DWI in terms of image quality, artifact reduction, and lesion detection, providing a robust basis for selecting the appropriate imaging modality for head and neck pathologies.

## Discussion

This study demonstrates that TSE provides superior image quality compared to EP-DWI in the head and neck region. The higher SNR and CNR in TSE images enhance the visualization of anatomical structures and pathological changes, while the lower prevalence of artifacts further improves diagnostic confidence [7,8]. These findings align with previous research. For instance, a study by Mori et al. [6] found higher SNR in TSE than in EP-DWI at 3T, suggesting that TSE may be particularly beneficial in high-field MRI settings for head and neck imaging [9].

Panyarak et al. supported these results by showing that high-resolution three-dimensional diffusion-weighted imaging using a turbo field-echo technique in the middle ear can reduce artifacts and improve image quality [10]. This aligns with the current study’s findings that TSE, as a non-EP technique, provides clearer images with fewer distortions. Additionally, a comparison of different DWI techniques indicates that TSE offers significant advantages over EP-DWI in terms of artifact reduction and image clarity, particularly in areas susceptible to magnetic field inhomogeneities [11].

The higher artifact presence in EP-DWI, especially motion artifacts, can be attributed to the longer acquisition times and sensitivity to patient movement. This limitation affects the overall diagnostic accuracy and reliability of EP-DWI in clinical practice [12]. For example, research by Yamashita et al. noted similar limitations in artifact prevalence and its impact on diagnostic accuracy [13].

The study’s findings suggest that TSE should be the preferred imaging modality for detailed anatomical assessment in the head and neck region. However, EP-DWI remains a useful adjunct, particularly in cases where diffusion characteristics are critical for diagnosis, such as in the evaluation of malignant tumors and stroke. Supporting this, research by Fujima et al. highlighted the complementary role of EP-DWI in evaluating perfusion-related parameters in head and neck squamous cell carcinoma [14]. Hence, TSE MRI provides superior image quality compared to EP-DWI for evaluating the head and neck region. The enhanced SNR, CNR, and reduced artifacts in TSE images result in better lesion detection and characterization [15]. While EP-DWI is valuable for functional imaging, its role should be complementary to TSE. Further studies with larger sample sizes and advanced imaging techniques may provide additional insights into optimizing MRI protocols for head and neck imaging.

The findings of this study are consistent with previous research, reinforcing the superior image quality and diagnostic advantages of TSE over EP-DWI for head and neck imaging. Verhappen et al. [16] highlighted similar benefits of the half Fourier single-shot turbo spin-echo (HASTE) technique over EPI, noting reduced artifacts and improved image clarity in head and neck cancer imaging, which aligns with our observations regarding TSE's performance. Additionally, Sumi et al. [17] demonstrated the efficacy of advanced diffusion techniques such as intravoxel incoherent motion (IVIM) in distinguishing between benign and malignant tumors, underscoring the clinical utility of high-quality diffusion imaging in head and neck pathology. These studies collectively advocate for the use of TSE as the preferred imaging modality in this region, emphasizing the need for further research to optimize MRI protocols for enhanced diagnostic accuracy and reliability.

This study has several limitations. It is a retrospective analysis, which may introduce selection bias and limit the control over imaging parameters and patient conditions. The study's sample size, while sufficient for preliminary conclusions, may not be large enough to generalize the findings across all clinical scenarios in head and neck imaging. The comparison focuses on specific MRI techniques, namely, TSE and EP-DWI, potentially overlooking other advanced imaging methods that could offer additional diagnostic benefits. Additionally, the study does not extensively evaluate the clinical impact of these imaging differences on patient outcomes, such as treatment decisions and prognosis. Further research with larger, more diverse populations and prospective designs is necessary to confirm these findings and explore their broader clinical implications.

## Conclusions

The study shows that TSE MRI offers superior image quality compared to EP-DWI for the head and neck region. TSE images have a higher SNR, CNR, and fewer artifacts, leading to clearer and more detailed anatomical visualizations. EP-DWI, though valuable for functional imaging such as assessing malignant tumors and stroke, has limitations due to longer acquisition times and sensitivity to movement. TSE should be the preferred modality for detailed anatomical assessments, and further research is needed to optimize MRI protocols for head and neck imaging.
